# Referral of Queensland women with endometrial cancer to genetic services

**DOI:** 10.1186/1897-4287-10-S2-A63

**Published:** 2012-04-12

**Authors:** YY Tan, J McGaughran, A Obermair, A Spurdle

**Affiliations:** 1 The University of Queensland School of Medicine, Herston QLD 4029, Australia; 2 Queensland Center for Gynaecological Cancer Research, Royal Brisbane & Women’s Hospital, Herston QLD 4029, Australia; 3 Genetic Health Queensland, Herston QLD 4029, Australia; 4 Genetics and Population Health Division, Queensland Institute of Medical Research, Herston Road, Brisbane QLD 4006, Australia

## 

Approximately 5% of all endometrial cancers are due to a hereditary disposition, and a majority of the cases were found in families with Lynch syndrome or hereditary non-polyposis colorectal cancer (HNPCC) syndrome. Whilst both men and women with Lynch syndrome have a significantly increased risk of developing colorectal cancer (18-69%), women face the additional lifetime risks of developing endometrial cancer – between 27% to 71% as compared to 2% in the Australian general population. Despite the increased risk, many eligible women who may benefit from genetic assessment are not being referred by their treating clinician. The purpose of this study is to evaluate the patterns of referral of women diagnosed with endometrial cancer to genetic services. Using the diagnostic, clinical and referral databases from three different sites, we were able to link data of endometrial cancer cases in Queensland from May 2005 to December 2007. We determined the percentage of women diagnosed with endometrial cancer who could have been referred based on at least one risk factor suggestive of Lynch syndrome, the percentage of women that were referred and the percentage of women that attended genetic services. The revised Amsterdam and Bethesda criteria guidelines were adapted and used to assess the appropriateness of referral. Preliminary results show that of the 955 new diagnosis of endometrial cancer, 29 women (3%) were referred and 17 (1.8%) attended. This suggests that women who may benefit from genetic assessment do not ultimately attend their scheduled appointment. The mean age of referral is 61.7 years, with seven women diagnosed under the age of 50. Of the seven women, three were found to be mutation carrier. The results will be used to improve identification and referral of women at risk of Lynch syndrome to genetic health services in Queensland, and to increase awareness of hereditary gynaecological cancer.

